# *Granulicatella* spp., a Causative Agent of Infective Endocarditis in Children

**DOI:** 10.3390/pathogens11121431

**Published:** 2022-11-28

**Authors:** Chiara Albano, Sara Bagarello, Salvatore Giordano, Maria Fiorella Sanfilippo, Calogero Comparato, Giuseppe Scardino, Valeria Garbo, Giovanni Boncori, Anna Condemi, Antonio Cascio, Claudia Colomba

**Affiliations:** 1 Department of Health Promotion, Maternal and Infant Care, Internal Medicine and Medical Specialties, University of Palermo, 90100 Palermo, Italy; 2 Division of Pediatric Infectious Diseases, “G. Di Cristina” Hospital, ARNAS Civico Di Cristina Benfratelli, 90100 Palermo, Italy; 3 Pediatric Cardiology Unit, “G. Di Cristina” Hospital, ARNAS Civico Di Cristina Benfratelli, 90100 Palermo, Italy; 4 Infectious and Tropical Diseases Unit, AOU Policlinico “P. Giaccone”, 90100 Palermo, Italy

**Keywords:** *Granulicatella*, nutritionally variant streptococci, infective endocarditis, pediatric

## Abstract

*Granulicatella* spp. are non-motile, non-sporulating, facultatively anaerobic Gram-positive cocci. Throughout the literature, these organisms have been referred to by several names, such as “nutritionally deficient streptococci”, “vitamin-B dependent streptococci” and “pyridoxal-dependent streptococci”, because of their fastidious nutritional requirements, which can often make culture isolation challenging. Known to be a member of the normal microbiota of the human oral cavity and urogenital and intestinal tracts, similar to other streptococci, *Granulicatella* spp. can cause bacteremia, sepsis and infective endocarditis. Considering the difficulty in growing this organism on culture medium, the fact that it is now included among the bacteria known to be responsible for culture-negative infective endocarditis suggests that its pathogenic role could be highly underestimated. Moreover, being considered such a rare causative agent, it is not a target of standard antibiotic empiric treatment. We present a rare case of *G. elegans* endocarditis in a young child and review the medical literature on *Granulicatella* endocarditis in the pediatric population, with the aim of sharing knowledge about this microorganism, which can be challenging for a clinician who is not familiar with it.

## 1. Introduction

*Granulicatella species*, formerly referred to as a Nutritionally Variant Streptococci (NVS), known as *Abiotrophia species*, are Gram-positive bacteria, which are part of the normal microbiota of the oral cavity and intestinal and genitourinary tracts [1,2] and have been found to be responsible for severe infections, especially in patients with predisposing factors.

Isolated cases of central nervous system infections [3,4,5,6,7], as well as keratitis [8], endophthalmitis [9], sinusitis, otitis media, prostatitis, cholangitis, arthritis [10,11,12] and osteomyelitis [13,14,15], have been reported, but the most significant clinical syndromes associated with *Granulicatella* spp. are bacteremia and endocarditis, the latter mainly observed in patients with a background of congenital cardiac disease [16,17].

Infective endocarditis (IE) from *G. elegan*s is rare in the general population and in children. Furthermore, it represents a diagnostic challenge because of the non-specific symptoms at presentation and difficulties in growing the organism on culture medium. We present a rare case of *G. elegans* endocarditis in a young child and review the medical literature on *Granulicatella* endocarditis, with the aim of highlighting atypical causative agents of endocarditis in children presenting with fever of unknown origin, particularly in those with a background of congenital cardiac disease.

## 2. Case Report

A 13-year-old Caucasian male presented to our hospital with a 1-month history of low-grade fever (Tc max 37.7 °C) and pharyngodynia, treated at home with macrolide antibiotics without improvement. His past medical history was significant for the presence of a peri-membranous interventricular septal defect and a high-velocity left-to-right shunt without any hemodynamic consequence. In the two months before admission, a mildly symptomatic infection with SARS-CoV-2 was reported.

The initial physical examination was unremarkable, except for a cardiac murmur 2-3/6 on the Levine scale, detected along the left sternal border. Cardiac tones and peripheral pulses were normal. The initial laboratory findings revealed only a slight increase in the C-reactive protein (subsequently reduced) with a normal blood count. Because of the persistent fever, a serological examination for CMV, a urine culture and the Widal-Wright test (to exclude Salmonellosis and Brucellosis) were performed with negative results. A chest X-ray and a subsequent CT scan showed a pronounced thickening of the interstitial fiber network of the lungs and signs of emphysema.

The echocardiography examination revealed the presence of an endocardial vegetation (1 × 0.8 cm).

At the same time, the growth of *G. elegans* was detected from the blood cultures previously carried out. The antibiogram showed resistance to macrolides and vancomycin. Intravenous antibiotic therapy with ceftriaxone and gentamicin was administered for 4 weeks, leading to complete clinical recovery of the patient, a major reduction in the size of the vegetation and negative follow-up blood cultures. The post-discharge cardiology follow-up led to the decision of performing a surgical procedure to correct the pre-existing interventricular septal defect.

## 3. Methods

A literature search on endocarditis and bacteremia due to *Granulicatella* spp. was performed on PubMed using the following keywords: *Granulicatella* AND (endocarditis OR bacteremia OR sepsis OR septicemia) AND (pediatric OR children OR child OR infant OR baby). No filters or language restrictions were applied to the results. Furthermore, all references listed were hand-searched, and a citation tracker was used to identify any other relevant papers. An article was considered eligible for inclusion if it reported data confirming systemic or cardiac localization of the disease, with the isolation of *Granulicatella* spp. from blood cultures of pediatric patients. The following epidemiologic and clinical variables were evaluated for each case: age; sex; clinical presentation; underlying conditions; blood culture results; echocardiography findings; the requirement of a surgical approach; and the therapy regimen adopted together with its length and outcome, which was considered good in patients responding to treatment without relapse (Table 1).

## 4. Results

From the literature search, we retrieved 10 articles published between 2001 and 2022, and only 4 [18,19,20,21] were eligible for inclusion in our review. Five cases of bacteriemia due to *Granulicatella* spp. in pediatric patients (three due to *G. adiacens* and two due to *G. elegans*) were reported in total, and two of them were complicated by endocarditis (rows 1 and 2 in Table 1), similar to our patient. The median age of patients was 6 years; four children were male, one was female, and one was unspecified. An analysis of underlying conditions revealed that the three children who developed endocarditis had a predisposing condition, such as congenital heart disease (CHD), or recent cardiac surgical procedures; for another patient, who also presented with an infundibular pulmonary stenosis and a history of cardiac catheterization performed 3 weeks before the onset of symptoms, an IE was considered possible despite a negative echocardiography, according to the modified Duke criteria (the patient fulfilled three minor criteria: fever, predisposing heart condition and microbiological evidence [19]). Clinical stigmata of endocarditis were absent in all patients, while the clinical presentation was particularly characterized by a low-grade fever, general malaise, asthenia, pharyngodynia, diarrhea and leg pain in one case. The laboratory findings showed only a slight increase in C-reactive protein levels. All patients had positive blood cultures, with three growing *G. elegans* and three growing *G. adiacens*. All the patients experienced a good outcome after antibiotic administration. The length of the therapeutic regimen varied from 4 to 6 weeks for endocarditis, while bacteremia was treated for a median time of 2 weeks. Surgical procedures were only required in one case, where a pre-existing heart condition, not a direct consequence of IE, was cured.

## 5. Discussion

The genus *Granulicatella*, which comprises three species (*G. adiacens*, *G. elegans* and *G. balaenopterae*), was recently identified after a taxonomic revision by Collins and Lawson [22]. It was historically classified as a Nutritionally Variant Streptococci (NSV), a group of microorganisms first described by Frenkel and Hirsch in 1961 [23] as being a new type of *Streptococci* exhibiting satellite formation around colonies of other bacteria, with specific growth characteristics, including thiol and pyridoxal nutrient requirements. To date, NSV includes two genera, namely, *Granulicatella* and *Abiotrophia* (which literally means “life nutrition deficiency”) [24], including the spp. *defectiva* and *para adiacens* [25].

*Granulicatella* and *Abiotrophia species* are natural inhabitants of the oral, intestinal and urogenital microbiota of humans [1,2]. Although rarely implicated in disease, they have been found to be responsible for isolated cases of central nervous system infections [3,4,5,6,7], keratitis [8], endophthalmitis [9], sinusitis, otitis, prostatitis, cholangitis, arthritis [10,11,12], osteomyelitis and lumbar spine infection [13,14,15,26], but the most frequently reported syndromes due to *Abiotrophia* and *Granulicatella species* are known to be bacteremia, septicemia and endocarditis [16,17].

As for endocarditis, NSV appear to be responsible for approximately 5% of all cases of streptococcal endocarditis in the general population, carrying a worse prognosis than infections with other streptococci [1,27].

The AHA published a scientific statement in 2015 that included and discussed newly available evidence about IE in children [28]. According to this statement, the frequency of endocarditis among pediatric patients appears to have increased over the past two decades, especially in those with congenital heart disease (to date, the predominant underlying condition for IE in children > 2 years of age in the developed world) but also in those without any identifiable risk factor. Such a trend is likely attributable to the improved survival of patients with structural heart defects (where even the corrective surgical procedure can become a long-term risk factor for postoperative IE [29]), as well as to the growing complexity of patient management in neonatal and pediatric intensive care (which has led to the increased incidence of neonatal IE, even in those with structurally normal hearts) [30]. Furthermore, children with no known risk factors can suffer from IE due to invasive procedures, such as dental procedures, bronchoscopy and tonsillectomy [31]. The IE clinical presentation in children is generally indolent with prolonged low-grade fever coupled with non-specific symptoms, such as fatigue, weakness, myalgia, arthralgia, headache, weight loss and diaphoresis. Moreover, the classical signs of extracardiac IE (e.g., petechiae, hemorrhages, Roth’s spots, Janeway lesions, Osler nodes, and splenomegaly) are extremely rare [32].

Both the cardiac examination and the echocardiography findings can be highly variable, depending on the type of presenting heart disease and the particular site of infection. The AHA, therefore, strongly recommends that blood cultures be carried out in young patients with fever of unexplained origin and a pathological heart murmur, a history of heart disease or a history of previous endocarditis [28]

The mortality rate of endocarditis ranges from 4% to 18%, and its complications include congestive heart failure; new or progressive valvular dysfunction; prosthetic valve dysfunction, including dehiscence; sinus of Valsalva rupture; myocardial dysfunction; the obstruction of conduits or shunts; pericardial effusion; and embolization [31,32].

The majority of organisms causing IE in children are known to be Gram-positive cocci, including the Viridans Group Streptococci (e.g., *Streptococcus sanguinis*, *S. mitis* group and *S. mutans*), staphylococci (both *S. aureus* and coagulase-negative staphylococci), β-hemolytic streptococci and enterococci. Less frequently, other organisms, such as the HACEK group (*Haemophilus* species, *Aggregatibacter* species, *Cardiobacterium hominis, Eikenella corrodens* and *Kingella* species), are implicated [30].

When a patient has clinical or echocardiographic evidence of IE but persistently negative blood cultures, a diagnosis of culture-negative endocarditis (CNE) is made. Because studies have revealed that CNE can account for 2.5% to 70% of all endocarditis cases among the general population varying by country [26,33], the inclusion of molecular diagnostics methods on surgical specimens (e.g., polymerase chain reaction (PCR)) in the modified Duke diagnostic criteria has been proposed [34].

CNE is commonly caused by recent antibiotic therapy or infection due to fastidious organisms, such as the HACEK group, as well as by the less common and, therefore, less known species, *Abiotrophia* and *Granulicatella* [28].

In our review, we only included three cases of IE due to G. species with full data available. However, references to a few other cases of IE due to NSV in patients who were less than 15 years old were also identified without the necessary data for their evaluation [20,35,36], suggesting that the role of *G.* species as a pathogen in endocarditis could be underestimated.

Further, the treatment of *Abiotrophia* and *Granulicatella* infections is complicated by a variable susceptibility to commonly used antistreptococcal antibiotics, such as penicillin and ceftriaxone, as well as by significant resistance to macrolides [37,38,39]. According to the AHA’s guidelines, “patients with IE caused by Abiotrophia, Granulicatella species, or streptococci with an MIC of >0.5 μg of penicillin per milliliter should be treated with the antibiotic regimen listed for enterococci”, which would be a 4 week treatment regimen of penicillin, ampicillin or ceftriaxone, combined with gentamicin for the first 2 weeks. Vancomycin is considered an effective substitute for children who are unable to tolerate β-lactam antibiotic drugs, in combination with a 4-week course of gentamicin. Blood concentration tests of vancomycin and gentamicin, as well as renal function tests, should be performed on a weekly basis because of the use of multiple nephrotoxic antibiotic drugs [28].

While an early surgical approach is required in the case of heart failure, progressive valve dysfunction, embolic phenomena and other life-threatening complications of IE [40,41], there are no data in children supporting a prophylactic surgical approach aimed at preventing complications or recurrencies. The decision for surgical intervention thus remains strictly individualized. In our case, the decision of a corrective surgical intervention was made in accordance with the AHA/ACC guidelines for the management of congenital cardiac disease in adult patients [42], as well as with the purpose of preventing recurrence.

## 6. Conclusions

*Granulicatella* spp. may represent a diagnostic challenge for clinicians who are not familiar with it, its specific nutrient requirements for culture and its specific antibiotic resistance patterns.

More specific and widely shared knowledge could help to outline its role among the other atypical causative agents of endocarditis in children.

## Figures and Tables

**Table 1:** Literature review of Endocarditis/Bacteremia described in pediatric population.

Authors	Age (y)	Sex	Underlying Condition	Clinical Presentation	Blood Culture	Echocardiography Findings	Peripheral Stigmata of Endocarditis	Surgery	Regimen	Outcome
Holloway V., et al., 2021 [18]	9	/	Repaired pulmonary atresia and Ebstein’s anomaly	Fever, night sweats and lethargy	Positive for *G. elegance*	No clear vegetation but increased stenosis of the right ventricle to pulmonary artery conduit	Absent	Not required	Benzylpenicillin (2 w) and ceftriaxone (4 w)	Full recovery
Maia De Luca, et al., 2013 [19]	7	F	Shone syndrome with a sub-total obstruction of the conduit	2 weeks of nocturnal fever (39); 2–3/4 systolic murmur and hepatosplenomegaly	Positive for *G. adiacens*	Filamentous vegetation (10 × 2 mm) on the homograft	Absent	Replacement of the conduct with a pulmonary valve homograft	Vancomycin and gentamicin (empiric, not effective). Then vancomycin and meropenem (6 w)	Full recovery
Maia De Luca, et al., 2013 [19]	5	M	Infundibular pulmonary stenosis. Cardiac catheterization performed 3 weeks before the onset of the fever	2-month history of fever, diarrhea and leg pain. General malaise, asthenia, anorexia, pale skin and a systolic murmur	Positive for *G. adiacens*	Negative The endocarditis was considered “possible” according to modified Duke criteria	Absent	Not required	Cefotaxime and gentamycin (empiric, not effective). Then ciprofloxacin and meropenem (4 w)	Full recovery
Leah Quartermain, et al., 2013 [20]	Newborn	M	None	Born via emergency C-section performed in a 29-year-old woman with fever (37.6 °C), after a diagnosis of fetal distress. Birth weight, 3.05 Kg; Apgar score, normal	Positive for *G. elegans*	Negative	Absent	Not required	Benzylpenicillin and amikacin (6 d)	Full recovery
Bizzarro MJ, et al., 2011 [21]	Newborn	M	None	Born from a repeated C-section performed in a 37-year-old woman. Birth weight, 2.85 Kg; Apgar score, normal; RDS diagnosed within an hour of birth	Positive for *G. adiacens*	Persistent pulmonary hypertension	Absent	Not required	Ampicillin and gentamicin (empiric, not effective). Then vancomycin and gentamicin (2 w)	Full recovery
Albano C., et al., 2022	13	M	Perimembranous interventricular septal defect.	1-month history of serotine fever (Tc max 37.7 °C) and pharyngodynia	Positive for *G. elegans*	Endocardial vegetation (1 × 0.8 cm)	Absent	Not required	Ceftriaxone and gentamicin (4 w)	Full recovery

## Data Availability

Not applicable.
